# A type II endoleak from an accessory renal artery treated with laser assisted, transgraft coil embolization: A case report

**DOI:** 10.1016/j.jvscit.2024.101598

**Published:** 2024-08-17

**Authors:** Marco Panagrosso, Katarina Björse, Timothy Resch

**Affiliations:** a Division of Vascular Surgery, Department of Cardiovascular Sciences, S. Anna and S. Sebastiano Hospital, Caserta, Italy; b Department of Vascular Surgery, Rigshospitalet, Copenhagen, Denmark; c Department of Clinical Medicine, University of Copenhagen, Copenhagen, Denmark

**Keywords:** Laser, Endovascular aneurysm repair, Accessory renal artery, Endoleak, Embolization, Coils

## Abstract

The main complications of coverage accessory renal artery (ARA) are renal infarction and potentially renal function impairment and type II endoleak if firm apposition to the aortic wall is not achieved. We describe the management of an ARA type II endoleak treated by laser-assisted, transgraft coil embolization (LATE). A 76-year-old patient underwent a computed tomography scan 4 years after endovascular aneurysm repair. The computed tomography scan showed an increase of sac diameter with type II endoleak originating from the left ARA as an effect of aortic neck dilatation. ARA embolization was performed successfully via fusion-guided laser in situ fenestration and standard coil placement.

The presence of an accessory renal artery (ARA) is quite common. It is estimated that between 15% and 30% of adults who undergo endovascular aneurysm repair (EVAR) have at least one ARA.^1^ An important aspect of EVAR planning is achieving an adequate proximal graft sealing. In case of short necks with an ARA of infrarenal origin, adequate sealing may require ARA coverage. Recent studies have indicated that no significant alteration of renal function is observed after ARA coverage.^1^^,^^2^ However, limited reports focus on type II endoleaks originating from ARA and the risk of late neck effacement and subsequent type IA endoleak this might cause. Simple ARA coverage by the stent graft seal is considered adequate and preventive selective embolization is not necessary. We describe the management of a type II endoleak case from an ARA treated with in situ laser fenestration-aided coil embolization. The patient gave consent for publication details and images of his case.

## Case report

**Fig 1:**
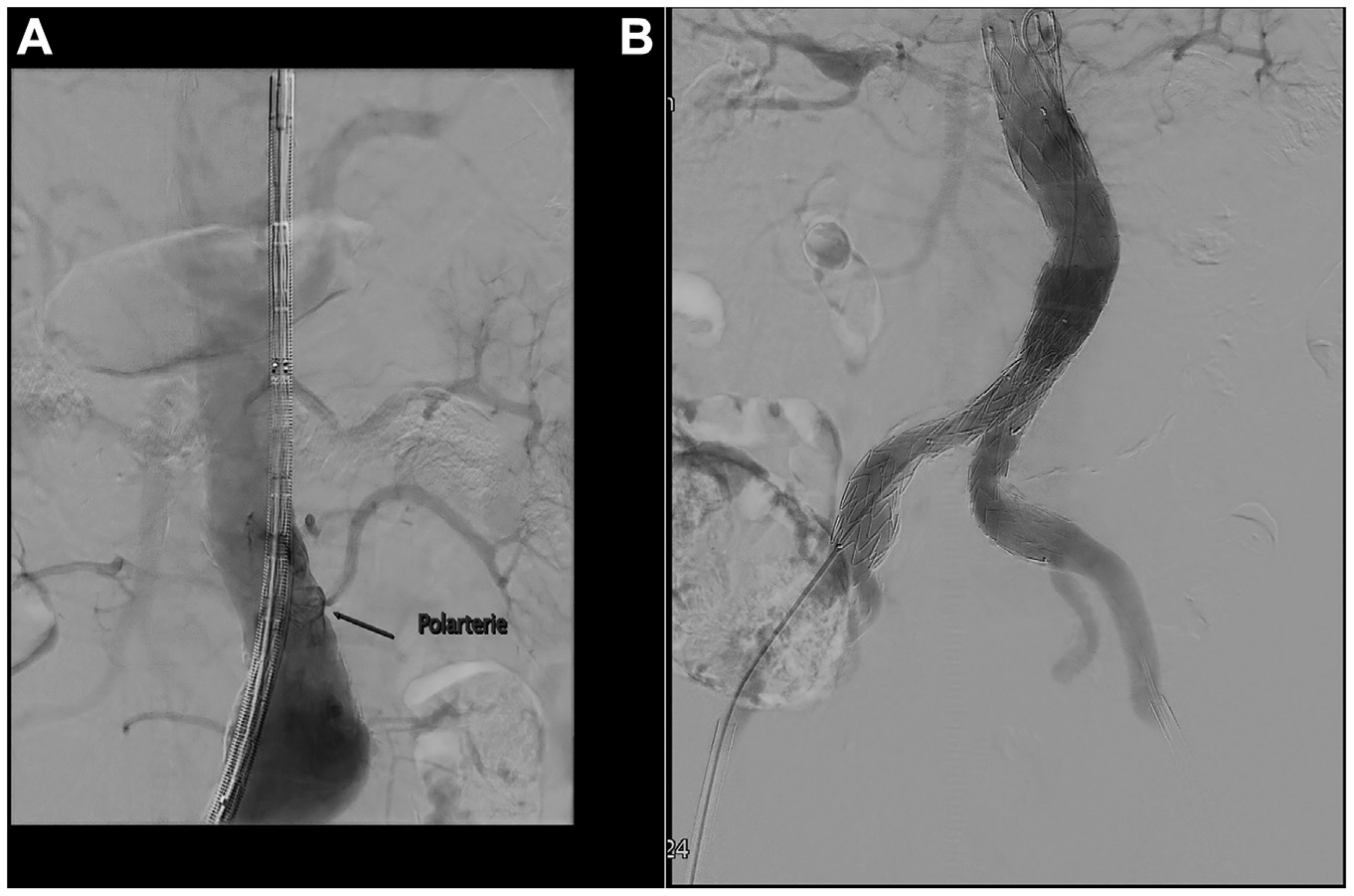
**(A/B)** Standard endovascular aneurysm repair (EVAR) **(B)** with accessory renal artery (ARA) coverage **(A)**.

A 76-year-old patient with a history of hypertension, diabetes and renal disease (golemerular filtration rate of 38 mL/min) underwent EVAR in November 2018 for a 58-mm aneurysm with a 25-mm neck diameter of the infrarenal abdominal aorta with coverage of a left ARA. The graft used was a Cook Zenith Alpha (Cook Medical, Bloomington, IN) (bifurcated main body diameter was 30 mm, left 20 mm iliac limb, right 24 mm iliac limb) (Fig 1, *A*).

The surgery was uneventful and final imaging showed no signs of any endoleak (Fig 1, *B*). During in-hospital stay, his renal function remained stable.

**Fig 2:**
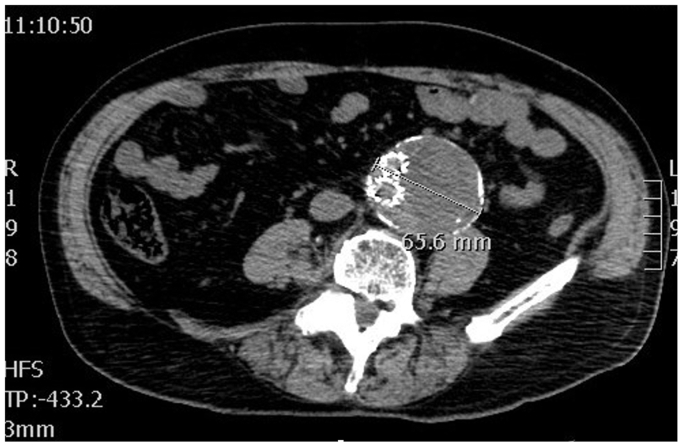
Computed tomography (CT) control scan after 1 year.

**Fig 3:**
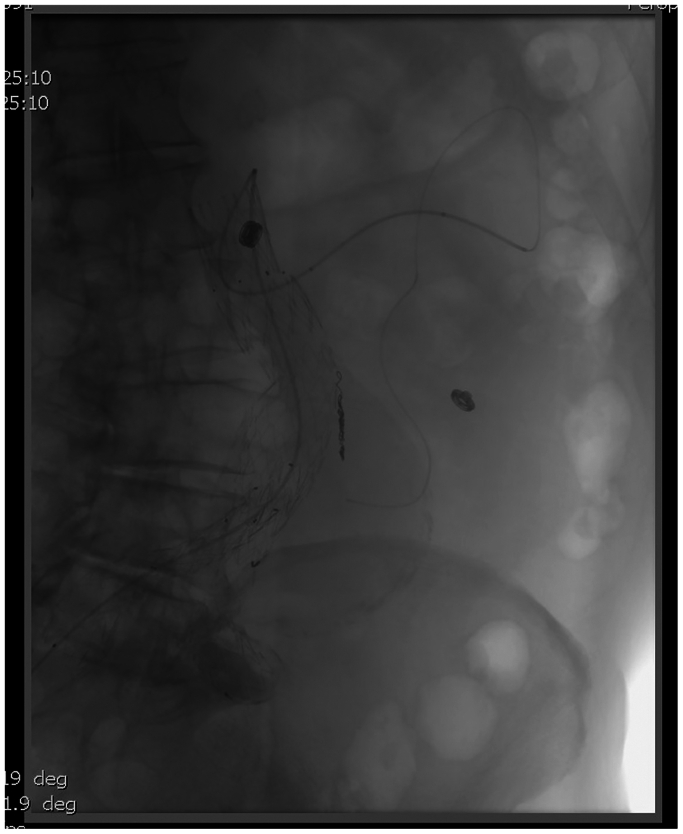
Coils embolization of the inferior mesenteric artery.

Subsequent computed tomography (CT) control, 3 years after EVAR, showed an abdominal aortic aneurysm expansion to 65 mm with a type II endoleak originating from the inferior mesenteric artery (Fig 2). Therefore, transarterial coil embolization of the inferior mesenteric artery was performed (Fig 3).

**Fig 4:**
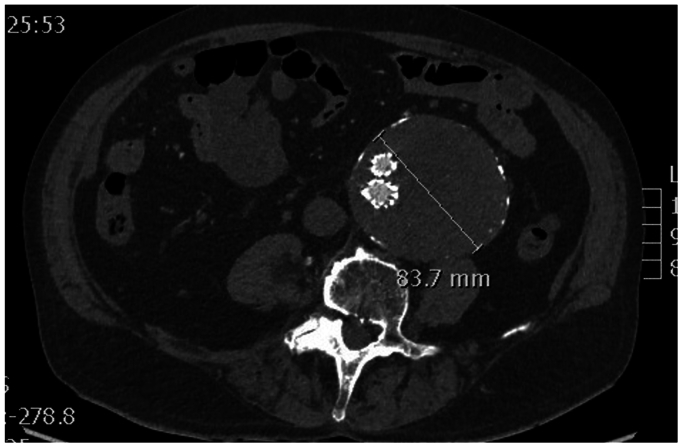
Computed tomography (CT) control scan after 1 year from inferior mesenteric artery coiling.

However, at further CT control at 1 year the residual sac continued to grow, reaching 83 mm (Fig 4) with evidence of a new type II endoleak originating from the left ARA secondary to proximal neck dilatation. However, because a type I endoleak could not be visualized on CT angiorgapy, contrast-enhanced ultrasound examintion, or angiography, we decided to attempt treatment of the accessory renal first considering the patients renal function and the complexity of a fenestrated cuff procedure. For this reason, we opted for embolization of the ARA, performed via laser in situ fenestration of the graft.

**Fig 5:**
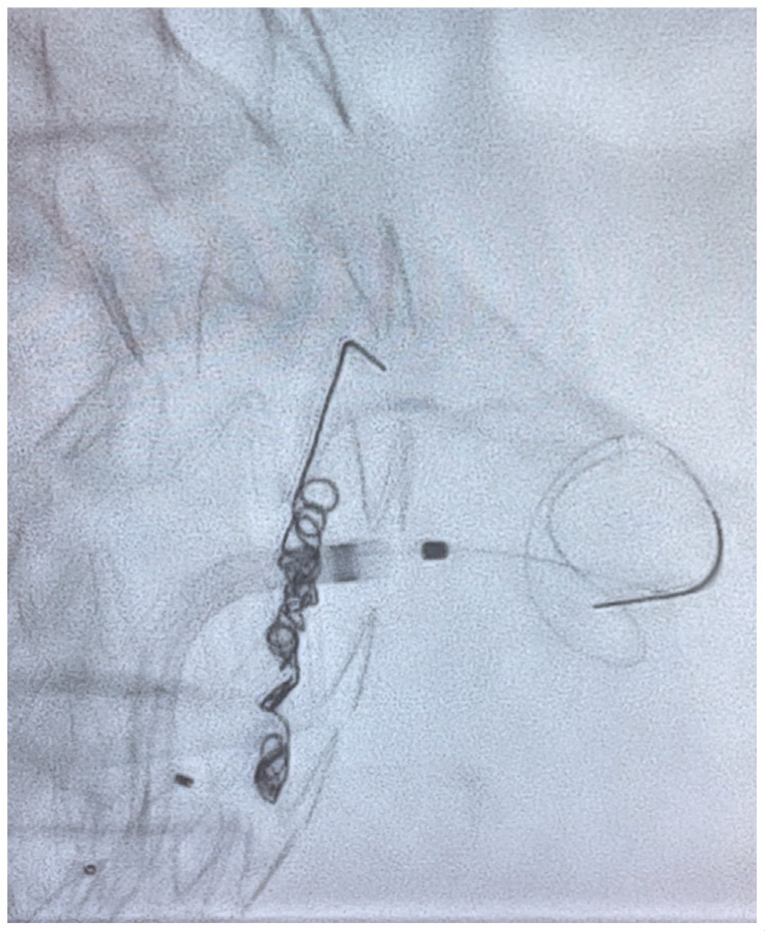
Laser in situ fenestration.

Percutaneous access of the right common femoral artery was performed under general anesthesia. A 7.5F APTUS TourGuide steerable sheath (Medtronic, Santa Rosa, CA) was used to guide a 0.014 laser probe (Philips laser system, Best, the Netherlands) to the target vessel ostia based on image fusion guidance. Maintaining a 90° angle with the steerable sheath and confirming accurate position of the laser probe by perpendicular C arm projections an in situ fenestration was performed. The ARA was catheterized with a Progreat Micro Catheter and a 0.018″ Terumo (Franklin Township. NJ) advantage guidewire (Fig 5). Three coils were released (Azur Detachable Coil 3 × 10 mm, Terumo, Japan). The artery was 2,5 mm, we aimed to oversize coils by 20$ to 40%. After removal of the microcatheter, a Medtronic aortic cuff (33 × 70 mm) was deployed across the fenestration, inside the 30-mm Zenith Alfa device to allow >10% interference fit, and post dilated with a Coda Balloon (Cook Medical Inc, Bjævreskov, Denmark). Access closure was performed with two Perclose ProStyle (Abbott, Abbott Park, IL) which had been applied in a preclose fashion at the start of the case. A total of 10 mL of contrast (Omnipaqe 140 mgI/l) was used for the procedure owing to renal insufficiency (golemerular filtration rate of 24 mL/min and creatinine of 2.56 mg/dL).

After 2 days, the patient was discharged without complications. At the 6-month follow-up there was continued abdominal aortic aneurysm growth despite radiologically well-embolized ARA. Continued growth without a visible endoleak after 1 year led to decision to treat with a four-vessel fenestrated proximal cuff extension and an iliac limb extension on the left side. The fenestrated extension cuff was 36 mm in diameter, thus allowing a 15% oversize. At the end of procedure a cone beam CT was performed. This procedure went well and no endoleak was visible on the final angiogram. The patient is now scheduled for Zenith branch iliac endovascular graft after new contrast-enhanced ultrasound examintion indicates a type IB leak from the right side.

## Discussion

The approach to abdominal aortic aneurysm surgery has shifted toward endovascular technique rather than open surgery in suitable patients. Anatomical suitability, including the length of the proximal neck and the presence of an ARA when originating from the infrarenal neck,^3^ must be considered.

The main complications to consider when performing EVAR including ARA coverage are renal infarction with subsequent renal function impairment and the potential for a type II endoleak.^2^ Such a type II endoleaks might contribute to neck dilatation secondary to aneurysm enlargement or in fact recur during late follow-up owing to primary neck dilatation owing to progression of disease.

The risk of renal function loss owing to covering of the ARA is often discussed. Several studies and systematic reviews have shown that, although there may be a risk of a potential infarction of the kidney, this does not lead to critical changes in renal function.^1^^,^^2^ However, many authors suggest that an ARA of >3 mm should be spared if possible.^4^ Several endovascular procedures have been developed and can be proposed to spare the ARA, including the use of fenestrated or branched endografts.

Type II endoleaks are the most frequent complications after EVAR, affecting approximately 20% of cases. Although most of them undergo spontaneous resolution, chronic type 2 leaks can lead to sac enlargement potentially and the risk of aneurysm rupture.^5^^,^^6^ There is far less evidence regarding the pathophysiology of type II endoleaks from the ARA. Clinically, it seems perhaps more aggressive than lumbar endoleaks with a lower rate of spontaneous closure. One possible mechanism might be an increase in pressure in the uncovered renal artery with subsequent increase in covered ARA, acting as an inflow.^4^ ARA type II endoleak might also cause localized neck dilatation with subsequent loss of endograft seal.

Many techniques are available for the treatment of type II endoleak, including open surgery or laparoscopic ligation of branches that perfuse the sac. Endovascular approaches are often favored and are usually preferred. Standard endovascular approaches involve transarterial, translumbar or transcaval embolization.^7^ Recently, the feasibility of endovascular laser fenestration of the endograft has described for different EVAR purposes such as revascularization of the left subclavian artery during TEVAR and to revascularize visceral vessels during emergent endovascular thoracoabdominal repair.^8^ Laser-assisted embolization of the aneurysm sac or lumbar arteries have also been described at international conferences. We decided to use the laser-assisted, transgraft coil embolization (LATE) technique to treat a type II infrarenal ARA endoleak with good postprocedural result. Using fusion imaging also allowed us to minimize the amount of contrast medium as well as guide the correct laser position during the procedure.

## Conclusions

This report is a description of successful LATE of type II endoleak owing to an ARA. We suggest that the LATE technique may be a valid alternative in treating these rare patients. More studies are needed to prove the efficacy and safety of this approach.

## Disclosures

None.
